# The Lives of Young Fathers: A Review of Selected Evidence

**DOI:** 10.1017/S1474746415000470

**Published:** 2016-01

**Authors:** Carmen Lau Clayton

**Affiliations:** School of Sociology and Social Policy, University of Leeds E-mail: C.Lau-Clayton@leeds.ac.uk

**Keywords:** Fatherhood, young fathers, family, service provision, parenting

## Abstract

While young fathers have been neglected in social research in the UK, over the past fifteen years a small but growing body of empirical evidence has emerged across a range of studies. This review article draws selectively on this literature to document the characteristics of young fathers in the UK and their lived experiences. It presents compelling evidence for the desire of young fathers to be engaged as parents, despite the sometimes multiple challenges that they face. The article begins with a demographic profile of young fathers and documents what is known of young fathers’ relationships with their children, the child's mother and wider kin. It goes on to consider a range of practical issues facing young fathers. The article concludes with a consideration of young fathers’ support needs and experiences of professional support, drawing out the implications for policy and professional practice.

## Introduction

Concerns about youthful fertility are not new. In popular discourse young parents are often portrayed as being irresponsible, ignorant and as a threat to the social order (Duncan, 2007). Mothers tend to be viewed as vulnerable, lone and morally suspect, while young fathers are frequently considered to be absent, no use, criminal and socially excluded (Johansson and Hammarén, 2014). In the past, teenage parenting research had primarily focused on young mothers, while young fathers were largely ignored (Turner, 2004). The lack of young fathers’ accounts has been attributed to access difficulties, uncooperative attitudes by gate-keepers and the unwillingness of young men to take part in research due to the legal implications of underage sex, or perceived negative attitudes towards them (Reeves, 2006). Young fathers can also be ‘hard to reach’ if mothers are reluctant to involve them during the pregnancy, birth or the child's upbringing, and if the pregnancy was unplanned (Ferguson and Hogan, 2004).

Although young fathers are an under-represented research group, academic interest in fatherhood can be traced back to the 1970s in both the US and the UK. A rich and diverse body of work has emerged from the 1990s onwards, in line with changing patterns of partnering and parenting (Lewis, 2000; Marsiglio *et al*., 2000). Over the last fifteen years, young fatherhood research has also grown in both countries. A large proportion of this research stems from America, and focuses on young, disadvantaged African-American and Latino fathers, where higher rates of early parenthood are most evident (Wei *et al*., 2002), but a more varied intercontinental context is consistently emerging. Studies have explored a range of issues, including contextual and behavioural predictors of young procreation (Miller-Johnson *et al*., 2004); the impact of young fatherhood on personal outcomes (such as education and income); fathers’ interest and involvement with their children (Speak, 2006); barriers and facilitators to fatherhood participation (Sheilds and Pierce, 2006); parenting skills (Nylund, 2006); relationships with the baby's mother (Reeves *et al*., 2009) and grandparents (Neale and Lau Clayton, 2014); and young men's engagement with professional services (Speak *et al*., 1997a). With one or two exceptions (for example, Quinton *et al*., 2002; Berrington *et al*., 2005; Kiernan, 2005; Shirani, 2015), this research is cross sectional; very little research evidence exists of a longitudinal nature, and most of this is statistical evidence from large-scale surveys, rather than qualitatively driven enquiry. This review article draws selectively on this literature to document the characteristics of young fathers in the UK and their lived experiences across a number of domains. In the process, the review highlights the heterogeneous nature of young fathers and the importance of understanding the lived experiences of young fatherhood within policy and practice.

## Demographic information

The under-eighteen conception rate in England and Wales is at its lowest since 1969. The estimated number of conceptions to women aged under eighteen was 24,306 in 2013, compared to 27,834 in 2012, which signifies a considerable decrease from the previous year (Office for National Statistics, 2015). Regional differences can be seen within these statistics. For example, in the north-east of England 30.6 per 1,000 women are aged fifteen to seventeen, in comparison to the south-east where 20.5 per 1,000 women are aged fifteen to seventeen (Office for National Statistics, 2015). Although teenage pregnancy has steadily decreased, pregnancy rates remain the highest in Europe (Public Health England 2014), while the number of pregnancies and births outside of marriage has also gradually decreased; in 1991, 18.2 per cent of UK teenage births occurred inside of marriage compared to 2.2 per cent in 2013 (Family planning Association, 2014).

For some, this may indicate a breakdown in parental relationships and suggest absentee fathers. However, in contrast to popular views which portray young fathers as feckless, promiscuous and uncaring (Barker, 2005), research consistently shows that many young fathers are committed to both mother and child, and are keen to have an active fathering role during and beyond the pregnancy (Quinton *et al*., 2002; Lohan *et al*., 2011). In one study, up to 80 per cent of young couples conceived in an ongoing relationship (Gates and Byrom, 2008). Furthermore, studies have found that 39 per cent of the young fathers in the sample lived with the teenage mother during the pregnancy (Kiernan, 2005), and at the time of the birth, two-thirds to three-quarters of young fathers are in a relationship with the mother (Kiselica, 2008). In the UK, 78 per cent of babies who are born to teenage mothers are registered in both their parents’ names (Fatherhood Institute, 2011), arguably demonstrating a high level of commitment to the child, regardless of the parent's relationship/marital status. Many young parents do eventually go on to marry, and the likelihood of adolescent parents marrying increases if they cohabit (Fagan *et al*., 2007).

## Age profile

Young fathers are commonly defined as those under the age of twenty-five (Fatherhood Institute, 2013). Due to common perceptions that individuals should postpone family life until their early thirties, young parents are typically positioned as an anomaly, whose early entry into parenthood is seen to violate social norms around individualised plans for education and employment (Duncan, 2007). In the UK, the average age for becoming a first time father outside of marriage is twenty-seven years (Office for National Statistics, 2015), and so in this context young parents are portrayed as deviating from normative life course practices. For babies who are born to teenage mothers, and where births are jointly registered, about a quarter of young fathers have been identified as aged twenty and under; in almost half the cases, fathers are aged twenty to twenty-four, and in one in six cases, fathers are aged twenty-five to twenty-nine (Hall and Hall, 2007). The children of teenage mothers often have fathers who are in their twenties (Dudley, 2007). Younger fathers are less likely to be involved or to stay involved compared to young adult fathers at the older end of the age spectrum (Kiselica, 2008). One study found 20 per cent of seventeen-year-olds are still involved nine months after the birth of their child, compared to 65 per cent of eighteen- to nineteen-year-olds, 56 per cent of twenty- to twenty-one-year-olds and 76 per cent of twenty-two to twenty-three-year-olds (Quinton *et al*., 2002).

Young fathers, like young mothers, are more likely to come from low-income families or suffer from social exclusion and poverty. Experiences include: living in areas with high unemployment rates, homelessness, living in care, history of drug and alcohol misuse in the family and poor educational outcomes (Swann *et al*., 2003). Incidences of teenage pregnancy may be more prevalent amongst certain ethnic minority groups, with Caribbean, Pakistani and Bangladeshi women more likely to become teenage mothers than white women (Berthoud, 2001). Research also suggests that early fatherhood is relatively common among men of African Caribbean heritage within both a British and an American context (Wei *et al*., 2002). Cultural attitudes and beliefs have been cited as possible precursors for early parenthood within minority community groups, demonstrating that beliefs around young fathering are constructed and understood differently across and within societies, and young parenthood is not necessarily considered problematic or undesirable (Arai, 2009). As Bonell (2004) argues, the view of young parenting as a major social problem is relative in terms of time, context and location. To date, little is known about the sexual practices and attitudes of British ethnic minority teenagers, or the ways in which potential risks or protective factors operate amongst them, indicating the need for further research on young minority ethnic parents (Teenage Pregnancy Unit, 2005).

From the available demographic information, young fathers cannot be seen as a homogenous group. The constitution of fatherhood, fathering practice and identities can be highly variable and are affected by individual and overlapping factors such as socio-economic background; ethnicity; opportunities for education, training and employment; residency type; relationship status with the baby's mother; support from maternal and paternal grandparents; and future trajectories (Neale and Lau Clayton, 2011).

## Relationships: young fathers and their children

When embarking on the journey of parenthood, young parents are said to be ‘positively ambivalent’, that is, they do not actively plan pregnancy but are open to the idea of having a child and welcome this when it happens (Alexander *et al*., 2010). The bond between a father and child may begin to develop during the pregnancy (Mansi, 2013), and upon the child's arrival young fathers often feel a strong emotional commitment to their children (Neale and Lau Clayton, 2011). Some young fathers report the benefits of their youthful age with regard to the physical aspects of childcare and play (Mansi, 2013), and many described parenthood as an accomplishment, a source of pride and responsibility, and a potential source of giving and receiving love (Ayoola *et al*., 2010). In one study, over 75 per cent of young fathers saw their children every day, despite many being the non-resident parent (Kiselica, 2008), indicating that young fathers want to be ‘hands on’ with their offspring, and be involved with activities such as food preparation, feeding, nappy changes, play time and visits to the doctor when illnesses occur (Ross *et al*., 2010).

Emotional availability is regularly cited as an important feature of modern fatherhood, often described as ‘being there’. ‘Being there’ can be described as a father who cares for his children and is emotionally close to them, an aspect of their parenting that sits alongside their traditional provider role (Neale and Lau Clayton, 2011). A young father's self-esteem may be affected by his inability to provide for his family. However, the potentially damaging effects of being out of work can be mitigated by a young father's significant practical involvement in the child's life (Paranjothy *et al*., 2009). The narrative of ‘doing my best’ has been highlighted as an alternative indicator of good fatherhood from young men's accounts (Shirani, 2015). Where there are high levels of father involvement, young men report higher levels of competence, satisfaction and investment in the paternal role (Rouch, 2005).

In terms of child contact times, some young fathers report this to be inadequate and express dissatisfaction with the extent of their paternal involvement (Centre for Research on Families and Relationships, 2009). Young fathers who do not engage with their children are reported to be distressed by their lack of involvement (Osborn, 2008). Where fathers express disinterest in their offspring, this is mainly associated with financial insecurity or uncertainty over the skills required for childcare, particularly for babies and younger children (Rhein *et al*., 1997). Stereotypes of ‘feckless’ fathers are arguably unhelpful and unrepresentative of young fathers’ behaviours and intentions when considering their engagement with their children.

Some political commentators (for example, Ian Duncan-Smith, 2011) have correlated young parenthood with the intergenerational transmission of poor parenting and welfare dependency. However research has shown that many young men want to be ‘better’ fathers than their own fathers were, and fulfil their paternal role in a contrasting manner to the one they had experienced (Tan and Quinlivan, 2006). Young fathers often cite the lack of a father figure in their own upbringing as the motivation for a desire to ‘be there’ and help ensure a more productive future for their child (Neale and Lau Clayton, 2011; Neale and Davies, 2015). The birth can encourage young fathers to face up to their responsibilities and to alter their reckless behaviour (for example, acts of alcohol and substance misuse; Reeves *et al*., 2009). Seen in such a light, parenthood may be seen as more of an opportunity for young men rather than a catastrophe as portrayed within official documents (Duncan, 2007).

## Relationship with the mother

The quality of the young father's relationship with the baby's mother and with grandparents also plays a vital role in terms of fatherhood engagement. In fact, it has a direct bearing on his paternal identity (Gavin *et al*., 2002). Where close father–mother relationships exist, fathers’ participation in childcare tends to increase and fathers spend more time with their children (Jaffe *et al*., 2001). Shifts in the gendered nature of parenting, in relation to social changes and attitudes, have been said to create a diverse range of parenting roles for mothers and fathers, which are no longer bound by traditional gender norms and responsibilities. Although some authors argue that, for the most part, parenting remains as a largely gendered activity (Dermott, 2008), such changes have created more opportunities for fathers to be involved and demonstrate alternative fatherhood practices to the breadwinner model.

Those who are separated from the mother of the child, or have not been in an established relationship beforehand, tend to have lower levels of paternal involvement compared to young fathers who are partnered (Beale, 2009). When young parents are in conflict with one another, the father may find it difficult to negotiate and stay in dialogue with the mother. This often leads to the young man backing away from the mother and child and becoming inconsistently involved (Nylund, 2006). Problems with the mother of the child are commonly cited as a barrier to the engagement of young fathers with their children. Young mothers may block access due to feelings of distrust or wariness of the baby's father, negative perceptions of the young man's parenting abilities and/or the formation of new romantic relationships by either parent (Reeves *et al*., 2009). Studies suggest that mothers’ and fathers’ successive partnerships and the arrival of additional children are associated with a decline in active fathering of any previous children (Smeeding *et al*., 2011). Currently there is a dearth of research that follows young fathers into the post-separation period, and much is unknown about family life after such a transition.

Although young fathers may have poor relationships with the mother of the child, and may not be co-resident with their children, they may still wish to be involved as parents (Neale and Lau Clayton, 2014). Young fathers can apply for a court order if they wish to improve child contact arrangements. In the past, young fathers could obtain legal aid for this process if they were on low incomes or had limited savings. However, since 2013 individuals can no longer apply for such support in child contact cases, unless there are issues of domestic violence and abuse (Legal Aid, Sentencing and Punishment of Offenders Act, Great Britain, 2012). Consequently, a larger number of young fathers have to navigate the complex court system independently (Lammy, 2013).

## The older generation

Research has indicated that maternal and paternal grandparents can play a central role in families, especially when parents are young themselves. The provision of kin care by grandparents may be particularly important for young parents, as those living in disadvantaged circumstances may have few resources to bring to parenthood and need on-going care themselves (Swann *et al*., 2003). Grandparents are often the main providers of practical, emotional and financial support for their children and new grandchildren (Neale and Lau Clayton, 2014). Positive relationships with grandparents and their acceptance of the grandchild are linked to increased fatherhood participation and care (Sheilds and Pierce, 2006). Conversely, the role of the maternal grandmother as gate-keeper is well documented within the literature, and grandparents may control, restrict or even block contact between a young father and his child (Neale and Lau Clayton, 2011).

For young parents who are dependent on, and who also live with, their respective parents, decisions about the care of the child have a propensity to run vertically down the generations, rather than horizontally between the young parents themselves (Neale and Lau Clayton, 2014). This pattern can be counterproductive to the efforts of young men to engage with their children and hinder their confidence levels. Young fathers’ accounts demonstrate that grandparental involvement can be seen as both a gift and a curse (Smith-Battle, 1996). Consequently, maintaining the balance between support, interference and neglect can be a difficult and complex matter (Neale and Lau Clayton, 2014).

## Practical issues

Trying to establish an active and consistent fathering role can present a range of practical challenges to young fathers. These relate to the basic provisioning of a family: finding a home, a job and securing financial resources (Reeves *et al*., 2009; Cundy, 2012). These make a significant difference to young parents’ lives and affect their relationships with their children and children's outcomes (Scott *et al*., 2012). Young men commonly report difficulties in securing a place of residence, which prevents them from having access to, and involvement with, their children (Berrington *et al*., 2005). Living in shared accommodation or in single hostels or bedsits may not be compatible with the needs of children. In these circumstances, mothers are likely to restrict contact for the father (Berrington *et al*., 2005). Securing social housing is also a challenge; since young fathers are not defined as the primary carer, their housing needs are seen as a lower priority. Where they are allocated social housing, young fathers may be placed in areas of isolation away from their family and friends through council accommodation procedures and housing shortages (Berrington *et al*., 2005; Alexander *et al*., 2010). Many young fathers lack financial resources and means of transport to be able to travel to see their child regularly and maintain informal support networks, potentially ‘isolating’ the young person in their new abode (Speak *et al*., 1997b). Research has found that young parents also move more frequently in comparison to older more settled parents, due to housing problems, fragile relationships with significant others and instances of ill health (St Michael's Fellowship, n.d.).

## Employment and finances

Young fathers frequently face a greater number of economic and employment challenges in comparison to older fathers; strong links have been reported between young parenthood and a lack of participation in education, training or employment, increasing the risks of persistent poverty and economic insecurity (Swann *et al*., 2003; Lemay *et al*., 2010). Longitudinal studies show that men who become fathers at a young age (under twenty-three) are twice as likely to be unemployed at age thirty than men who became fathers aged over twenty-three, even after taking account of deprivation (Centre for Social Justice, 2013). For young fathers who are employed, those under the age of twenty-three tend, on average, to have the least earning potential in comparison to older fathers in the twenty-four to thirty age bracket (Berrington *et al*., 2005).

Employment opportunities for young fathers may be improved by re-entering the education system. However barriers include a lack of childcare, inadequate and inflexible childcare provision and young parent's concerns about stigmatisation from peers and staff (Gates and Byrom, 2008). If long-term educational and employment goals are difficult for young men to attain, they may view paternity as a source of prestige instead (this may be particularly the case for those from disadvantaged families; Marsiglio *et al*., 2000). On the other hand, some sources suggest that financial hardship can lead to disassociation and reduced participation in their child's life, even though the majority of young fathers wish for the opposite (Glikman, 2004). For most young parents, economic insecurity is reinforced through their dependence on welfare support. Longitudinal evidence from the 1970 British Birth Cohort Study (Berrington *et al*., 2005) suggests that post school age young fathers (under twenty) and young adult fathers (under twenty-three) are significantly more likely to receive welfare benefits than older fathers (25 percent, 30 percent and 15 percent, respectively).

## Young fathers’ support needs

As the above review shows, despite their aspirations to ‘do good’ and ‘be there’ for their children, young fathers may face considerable challenges and barriers to parenthood. For those living in disadvantaged circumstances, these challenges are all the greater, indicating the need for professional support and encouragement. When the right services are in place and good support is provided at the right time, poor outcomes for young parents and their children can be addressed (Hadley, 2014). Where the involvement of young fathers is supported at the antenatal stage and during the birth, they are more likely to maintain this involvement over time (Maxwell *et al*., 2012). However young fathers often report very negative experiences of midwifery and health visiting services, as they are seen as unsympathetic, judgmental and ill-informed about the lives of young fathers (Paranjothy *et al*., 2009). Many young men have specifically reported feelings of exclusion and marginalisation during their encounters with service providers (Roskill *et al*., 2008). Evidence suggests that some family services do not view engagement with, or pro-active support for, young fathers as a priority (Page *et al*., 2008). Fathers may be constructed as absent, no use, troubled or troublesome, in a variety of professional settings. This is not simply confined to child protection and surveillance services, that have a safeguarding remit, and that tend, inevitably, to view young fathers as both *at risk* and *as a risk:* a threat and danger to themselves, to women and children (Featherstone *et al*., 2007; Maxwell *et al*., 2012; Featherstone, 2013). It is a perception that has become ingrained within official orthodoxies and popular thinking on young parenthood (Duncan, 2007).

Young parents are often acutely aware of the dominant stereotypes surrounding early parenthood in society and this in itself can act as a barrier to their engagement with professional services. Many young fathers have expressed a desire to receive guidance from practitioners (especially for the first child) in the time leading up to the birth and afterwards (Ayoola *et al*., 2010), and, arguably, this key moment as they undergo a major life transition is when they are most open to learning new skills as legitimate clients of services (Neale and Lau Clayton, 2011). The evidence suggests a desire for knowledge and preparation regarding basic childcare activities (such as bathing, handling infants and feeding procedures; McDonnell *et al*., 2009) as well as negotiating relationships with the mother of the child post-birth (Ashley, 2011).

If a young father refrains from seeking out professional support due to negative responses from practitioners or discriminatory attitudes, they risk being labelled as irresponsible or absent (Featherstone *et al*., 2007). This may explain why some studies have labelled young fathers as ‘hard to reach’. However the evidence also suggests that services may be ‘hard to use’, rather than client users being ‘hard to reach’. As Murphy (2006, citied in Gupta *et al*., 2008) suggests, the term ‘hard to reach’ indicates that the problem lies within the group itself, as opposed to services’ approach to clientele. ‘Hard to reach’ also implies a homogeneous group in a way that belies the heterogeneous circumstances and characteristics of young fathers (Ayoola *et al*., 2010). By recognising the diverse nature of young fatherhood, policy makers and practitioners are able to frame service support from a different and more sympathetic perspective, one which is validated by the accounts of young men themselves. Engaging successfully with young fathers also requires a positive rethinking of such men. As Neale and Davies (2015: 3) suggest:

The challenge, then, is one of changing the culture of professional practice so that young fathers are no longer discounted as ‘hard to reach’, ‘disinterested’, or ‘risky’ but sought out and welcomed as clients with a valuable contribution to make.

When appropriate formal support is provided, this is linked to better psychological, emotional and economic wellbeing of young parents, better quality of parenting and may prevent serious case reviews from occurring (Hadley, 2014). Examples of good practice can be seen in a number of local authorities in terms of successfully engaging and working with young fathers (Davies and Neale, 2015). Well-funded and structured provision has been found to make a significant difference to the lives of young fathers and their children (Cundy, 2012). However, such support is confined to selected localities and they tend to be piecemeal and small scale, with limited funding set apart from mainstream services (Trivedi *et al*., 2009). Consequently provision for young fathers is fragmented and patchy with an over reliance upon ‘local champions’ (Neale and Davies, 2015). Fathers still do not generally feature as a specific policy area or a concern in their own right despite political rhetoric (Cundy, 2012). Young fathers need to be identified more effectively (Osborn, 2015), but tackling professional attitudes towards young fathers only forms one part of the solution. Services need to be better co-ordinated and resourced if service providers are to successfully reach out (Osborn, 2015).

## Concluding comments

Overall, this article indicates that young men's abilities to engage and sustain a fathering role are dependent on a number of complex and often interlinking factors such as education, training and employment opportunities, relationship status with the baby's mother, support levels from maternal and paternal grandparents, residence arrangements and access to formal service provision – many of which can become barriers for them to overcome.

With regards to formal service provision, timely access to appropriate support can help a young man's positive transition into parenthood, promote fatherhood engagement and is shown to be beneficial, regardless of the father's relationship status with the baby's mother. However, commentators argue that provision for young fathers is still lacking. While young mothers encounter universal provision, such as midwifery and health visiting, such systematic contact with young fathers continues to be limited. Much more needs to be done to support and promote a father inclusive culture within policy and professional practice. Listening to the voices of young fathers and understanding their lived experiences can help overturn negative perceptions of them as parents and thereby contribute to better service provision. As Lammy (2015: 11) observes, ‘Whilst Government policy alone cannot make better fathers, the UK needs to up its game when it comes to supporting them . . . A society of involved fathers is a better society for everyone.’
